# Strain-Specific Contribution of Eukaryotic Elongation Factor 1 Gamma to the Translation of Influenza A Virus Proteins

**DOI:** 10.3389/fmicb.2018.01446

**Published:** 2018-06-29

**Authors:** Shuhei Sammaibashi, Seiya Yamayoshi, Yoshihiro Kawaoka

**Affiliations:** ^1^Division of Virology, Department of Microbiology and Immunology, The Institute of Medical Science, The University of Tokyo, Tokyo, Japan; ^2^Department of Pathobiological Sciences, School of Veterinary Medicine, University of Wisconsin–Madison, Madison, WI, United States; ^3^Department of Special Pathogens, International Research Center for Infectious Diseases, The Institute of Medical Science, The University of Tokyo, Tokyo, Japan

**Keywords:** influenza virus, host protein, eEF1G, protein translation, PB2, PA

## Abstract

Influenza A virus exploits multiple host proteins during infection. To define the virus–host interactome, our group conducted a proteomics-based screen and identified 299 genes that contributed to virus replication and 24 genes that were antiviral. Of these genes, we focused on the role during virus replication of eukaryotic elongation factor 1 gamma (eEF1G), which is a subunit of the eukaryotic elongation factor-1 complex and known to be a pro-viral host protein. Using the CRISPR/Cas9 system, we obtained two clones that were defective in eEF1G expression. In both of these clones, A/WSN/33 (H1N1) virus growth and protein expression were significantly suppressed, but viral mRNA, vRNA, and cRNA expression were not reduced. However, the replication and protein expression of A/California/04/2009 (H1N1pdm) virus in both clones were similar to those in parental cells. We found that the PB2 and PA proteins of WSN virus were responsible for the eEF1G-dependent replication. Our data show that eEF1G plays a role in the translation of virus proteins in a strain-specific manner. Additional analyses may be needed to further understand the role of strain-specific host proteins during virus replication.

## Introduction

Influenza A virus possesses eight single-stranded, negative-sense RNAs as a genome (Palese and Schulman, 1976). Because viruses encode limited numbers of proteins in the genome, they depend on host cellular factors such as enzymes, lipid bilayers, and cellular machineries to replicate. To better understand the host–virus interaction during virus replication, eight genome-wide screens have been reported and each identified numerous host proteins that are required for virus replication (Brass et al., 2009; Shapira et al., 2009; Karlas et al., 2010; König et al., 2010; Ward et al., 2012; Su et al., 2013; Tran et al., 2013; Watanabe et al., 2014). Although these genome-wide screens of virus–host interactions identify candidate host proteins involved in viral replication, the roles of the candidate proteins in the various steps of the virus replication cycle require further analyses. Previously, our interactome analysis, followed by siRNA knockdown screening, found that eukaryotic elongation factor-1 gamma (eEF1G), which interacted with several virus proteins including PB2, PB1, PA, and NP, was involved in influenza A virus replication (Watanabe et al., 2014). Although down-regulation of eEF1G expression reduced virus propagation *in vitro*, it was not clear which step of virus propagation eEF1G is involved in.

**FIGURE 1:**
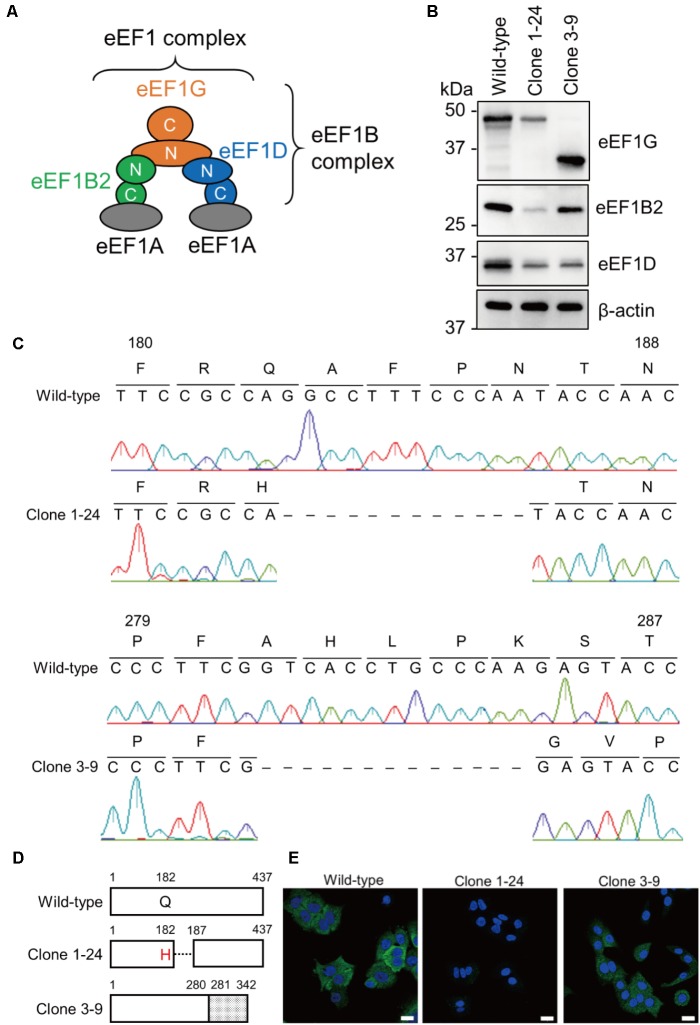
Acquisition of clones 1–24 and 3–9. **(A)** A model of the eEF1 complex. The eEF1 complex consists of two eEF1A molecules and an eEF1B complex. eEF1B2, eEF1D, and eEF1G comprise the eEF1B complex. **(B)** Expression of eEF1G, eEF1B2, and eEF1D in clones 1–24 and 3–9. Total cell lysate of each cell line was analyzed by western blotting; β-actin served as a loading control. **(C)** Nucleotide sequences of the eEF1G mRNA expressed in clones 1–24 and 3–9. The eEF1G mRNA sequence was obtained by RT-PCR, followed by Sanger sequencing. Corresponding amino acids are presented above the nucleotide sequences. Dashed lines indicate deletion of the corresponding nucleotides. **(D)** Schematic diagram of eEF1G expressed in clones 1–24 and 3–9. eEF1G in clone 1–24 had a single amino acid substitution at position 182, followed by a 4-amino acid deletion. eEF1G in clone 3–9 was composed of the N-terminal authentic 280 amino acids and C-terminal frameshifted 62 amino acids (gray). **(E)** Intracellular localization of eEF1G in clones 1–24 and 3–9. eEF1G (green) was detected by using a rabbit anti-eEF1G mAb, followed by the Alexa Fluor 488 anti-rabbit IgG. Nuclei (blue) were stained with Hoechst 33342. Scale bar: 20 μm.

The eukaryotic elongation factor-1 (eEF1) complex plays central roles in peptide elongation during eukaryotic protein synthesis (Riis et al., 1990; Le Sourd et al., 2006). The eEF1 complex consists of two functional parts, eEF1A and eEF1B complex (**Figure 1A**). eEF1A in its GTP-bound form binds and delivers aminoacyl-tRNAs to the ribosome (Carvalho et al., 1984). eEF1B complex, which is composed of three subunits (eEF1B2, eEF1D, and eEF1G), acts as a guanine nucleotide exchange factor (GEF), reactivating the inactive GDP-bound form of eEF1A to the active GTP-bound form (Janssen and Möller, 1988). The eEF1 complex is thought to exist as a pentamer composed of two eEF1A molecules, plus one molecule each of eEF1B2, eEF1D, and eEF1G (Mansilla et al., 2002). eEF1G holds both eEF1B2 and eEF1D at its N-terminus (Mansilla et al., 2002). eEF1B2 and eEF1D bind to eEF1A independently and both act as GEFs through a C-terminal catalytic domain (Janssen and Möller, 1988). The primary role of eEF1G may be to ensure the proper scaffolding of eEF1B2 and eEF1D in the eEF1B complex (Le Sourd et al., 2006), but its precise role remains unknown.

Here, we characterized the role of eEF1G during influenza virus replication to further understand the interaction between influenza A virus and its host during virus replication.

## Materials and Methods

### Cells

Human alveolar adenocarcinoma epithelial A549 cells were cultured in Ham’s F-12K medium (Wako) supplemented with 10% fetal calf serum (FCS) and antibiotics at 37°C in a humidified 5% CO_2_ atmosphere. Mardin-Darby canine kidney (MDCK) cells were cultured in Eagle’s minimal essential medium (MEM) supplemented with 5% newborn calf serum (NCS) and antibiotics at 37°C in a humidified 5% CO_2_ atmosphere.

### Viruses and Reverse Genetics

Influenza viruses, A/WSN/33 (WSN; H1N1), A/California/ 04/2009 (CA04; H1N1pdm), A/Perth/16/2009 (Perth16; H3N2), and their reassortant viruses were propagated in MDCK cells. All of the viruses used in this study were generated by use of plasmid-based reverse genetics, as described previously (Neumann et al., 1999; Yamada et al., 2010). The titers of the stock viruses were determined by use of plaque assays in MDCK cells. All viruses were sequenced to confirm the absence of unwanted mutations.

### Antibodies

Mouse monoclonal anti-M1 antibody (C111; Takara Bio), mouse monoclonal anti-NP antibody clone 2S-347/3 (available in our laboratory), rabbit monoclonal anti-eEF1G antibody (ab124994; Abcam), rabbit polyclonal anti-eEF1B2 antibody (10095-2-AP; proteintech), mouse monoclonal anti-eEF1D antibody (60085-1-Ig; proteintech), rabbit monoclonal anti-eIF3B antibody (ab133601; Abcam), rabbit polyclonal anti-SNRPA antibody (ab40689; Abcam), mouse monoclonal anti-β-actin antibody (A2228; Sigma), mouse monoclonal α-tubulin antibody (T6199; Sigma), HRP-conjugated anti-mouse IgG (GE Healthcare), and HRP-conjugated anti-rabbit IgG (GE Healthcare) were purchased from the sources indicated.

### Gene Editing

CRISPR guide RNA (gRNA) sequences were designed at the Genetic Perturbation Platform web portal site^1^. Two gRNAs for eEF1G, gRNA1 (5’-TGGTATTGGGAAAGGCCTGG-3’) targeting exon six and gRNA3 (5’-CCTTCGCTCACCTGCCCAAG-3’) targeting exon seven, were cloned into the pLentiCRISPR v2 vector (52961; Addgene). A549 cells were transfected with each pLentiCRISPR encoding the gRNA for eEF1G by using lipofectamine LTX reagent (Thermo Fisher Scientific, Waltham, MA, United States) and then selected by use of puromycin (1 μg/ml) to obtain stable transformants. Two weeks after selection, 46 and 11 clones were picked from the gRNA1- and gRNA3-transfected cells, respectively, and the expression of eEF1G from each clone was analyzed by western blotting, as described below. Total RNA isolated from each clone was reverse-transcribed with oligo dT primer and SuperScript III Reverse Transcriptase (Life Technologies, Carlsbad, CA, United States). PCR was performed with the forward primer eEF1G-Fw1 (5’-CACAGAGCTCACCATGGCGGCTGGGACCCTGTAC-3’) or eEF1G-Fw2 (5’-TGTTGTGGCTCTATAAGCAGGTTC-3’), and the reverse primer eEF1G-Rv1 (5’-CACAATCGATTCACTTGAAGATCTTGCCCTG-3’) or eEF1G-Rv2 (5’-TGAGTTCTTCAGGGAAGCGATAC-3’), by using KOD-FX (TOYOBO) as follows: after 2 min of denaturation at 94°C, samples were subjected to 30 cycles of amplification, consisting of 10 s at 98°C, 30 s at 50°C, and 90 s at 68°C, with a final additional extension step at 68°C for 5 min. The nucleotide sequence of the PCR product was determined by using the 3130xl Genetic Analyzer (Life Technologies, Carlsbad, CA, United States) and the Bigdye Terminator v3.1 Cycle Sequencing Kit (Life Technologies, Carlsbad, CA, United States).

### Establishment of Cell Lines Stably Expressing gRNA-Resistant eEF1G

To generate a plasmid for gRNA1-resistant eEF1G, the open reading frame of eEF1G possessing seven synonymous nucleotide mutations was cloned into pcDNA3.1 (pcDNA3.1/gRNA1-resistant eEF1G) by using standard PCR techniques. Clone 1–24 was transfected with pcDNA3.1/gRNA1-resistant eEF1G by using lipofectamine LTX reagent (Thermo Fisher Scientific, Waltham, MA, United States) and then selected by use of G418 (1 mg/ml) to obtain stable transformants. Two weeks after selection, 11 clones were picked and the expression of eEF1G in each clone was analyzed by western blotting, as described below.

### Coomassie Brilliant Blue Staining and Western Blotting

Samples were prepared with Tris-Glycine SDS sample buffer (Invitrogen, Carlsbad, CA, United States) and incubated for 10 min at 95°C. Denatured samples were loaded onto an Any KD Mini-PROTEAN TGX Gel (Bio-Rad, Hercules, CA, United States). Separated proteins in the gel were visualized by Coomassie brilliant blue (CBB) Stain One (Nacalai Tesque), according to the manufacturer’s instructions. For western blotting, the separated proteins were transferred to an Immobilon-P PVDF membrane (Millipore). The membrane was blocked with Blocking One Solution (Nacalai Tesque) for 30 min at room temperature. The membrane was then incubated with the indicated primary antibodies diluted in Can Get Signal Solution 1 (TOYOBO) for at least 12 h at 4°C, followed by incubation with secondary antibodies diluted in Can Get Signal Solution 2 (TOYOBO) for 1 h at room temperature. Signals were detected by using Chemi-Lumi One Super (Nacalai Tesque) and the ChemiDoc Touch Imaging System (Bio-Rad).

### Immunofluorescence Assay

The immunofluorescence assay was performed as previously reported (Yamayoshi et al., 2008; Yamayoshi et al., 2016), with some modifications. Briefly, wild-type A549 cells, clone 1–24 cells, or clone 3–9 cells were fixed with 4% paraformaldehyde and then permeabilized with 0.2% Triton X-100. After blocking, antigens were probed with the rabbit anti-eEF1G mAb, followed by Alexa Fluor 488 goat anti-rabbit IgG (Life Technologies, Carlsbad, CA, United States). Nuclei were stained with Hoechst 33342 (Life Technologies, Carlsbad, CA, United States). The cells were then imaged by using a laser-scanning microscope (LSM780 system, Carl Zeiss) and analyzed with Zen software (Carl Zeiss).

### Cell Proliferation Assay

Wild-type A549 cells, clone 1–24 cells, or clone 3–9 cells were seeded in 12-well plates at a density of 100,000 cells per well in Ham’s F-12K medium supplemented with 10% FCS. The number of cells in a well at the indicated time points was counted using a LUNA-FL Automated Fluorescence Cell Counter (Logos Biosystems). To analyze the expression of endogenous proteins, wild-type A549 cells, clone 1–24 cells, or clone 3–9 cells were seeded in 12-well plates at density of 300,000 cells per well in Ham’s F-12K medium supplemented with 10% FCS. At 24 h after seeding, cell lysates were prepared with SDS-sample buffer containing 50 mM dithiothreitol and were analyzed by CBB staining or western blotting as described above.

### Comparisons of Virus Growth *in Vitro*

Wild-type A549 cells, clone 1–24 cells, clone 3–9 cells, clone 1-24#1, and clone1-24#4 were infected with the indicated viruses at a multiplicity of infection (MOI) of 0.001 for WSN-based viruses or of 0.01 for CA04-based viruses. After incubation for 1 h at 37°C, the inoculum was removed and replaced with Ham’s F-12K medium supplemented 0.3% bovine serum albumin, TPCK (tosyl-L-phenylalanine chloromethyl ketone)-treated trypsin (1 μg/ml), and antibiotics. Infected cells were incubated at 37°C in 5% CO_2_. At the indicated times after infection, the virus titers in the cell culture supernatant were determined by means of plaque assays in MDCK cells.

### Viral Protein Expression in Infected Cells

Wild-type A549 cells, clone 1–24 cells, or clone 3–9 cells were infected with WSN or CA04 virus at an MOI of 10. At 3, 6, 9, and 12 hours post-infection (hpi), cells were lysed with SDS sample buffer containing 50 mM dithiothreitol. The samples were then analyzed by western blotting as described above.

### Strand-Specific RT-qPCR

To quantify vRNA, cRNA, and mRNA in infected cells, strand-specific RT-qPCR was performed as previously described (Kawakami et al., 2011). Briefly, wild-type A549 cells, clone 1–24 cells, or clone 3–9 cells were infected with the indicated virus at an MOI of 10. At 2, 4, and 6 hpi, isolated total RNA was reverse-transcribed using SuperScript III Reverse Transcriptase with influenza gene-specific tagged primers at the 5’ end, and qPCR was then performed using Thunderbird SYBR qPCR mix (TOYOBO) on an ABI PRISM 7900HT.

### Statistical Analysis

Data are shown as the mean ± SD (*n* = 3). The one-way or two-way analysis of variance (ANOVA) followed by Dunnett’s test was performed using GraphPad Prism software (v6.05). A *P*-value < 0.05 was considered significantly different. Two-tailed unpaired *t*-tests with Bonferroni correction were used to compare groups at the indicated timepoints. A *P*-value < 0.05 was considered significantly different.

### Data Availability

All data analyzed during this study are included in this article.

## Results

### Edition of the eEF1G Gene by the CRISPR/Cas9 System

To confirm the importance of eEF1G in influenza virus replication *in vitro*, we attempted to generate an eEF1G knockout cell line by using the CRISPR/Cas9 system. Wild-type A549 cells were transfected with two kinds of pLentiCRISPR encoding gRNA1 or gRNA3, targeting exon six or seven, respectively, of the eEF1G gene. A total of 46 and 11 clones were picked from the gRNA1- and gRNA3-transfected A549 cells, respectively, and were examined for eEF1G expression by western blotting. We failed to obtain any eEF1G knockout cells most likely because eEF1G is an essential gene for proliferation and survival in human cancer cell lines (Wang et al., 2015). Instead, we obtained two clones (1–24 and 3–9) with a defect in eEF1G expression. Expression of eEF1G in clone 1–24 was decreased compared with that in wild-type cells, whereas a shorter form (∼37 kDa) of eEF1G was detected in clone 3–9 (**Figure 1B**). To delineate the edited eEF1G, we determined the nucleotide sequence of the mRNA encoding eEF1G in each clone. We found that 12 or 13 nucleotides were deleted in the eEF1G mRNA of clone 1–24 or 3–9, respectively, resulting in an amino acid substitution (Q182H) and a 4-amino acid deletion or a frameshift after position 281 (**Figures 1C,D**). The antibody used to detect eEF1G was a rabbit monoclonal antibody against a peptide that corresponds to an area distant from the amino acids around positions 182–186 (Abcam; proprietary information). Therefore, the reduced reactivity of the mutant eEF1Gs detected with this antibody is likely indicative of the reduced level of mutant eEF1G expression or alteration in its structure. These results suggest that clones 1–24 and 3–9 have a defect in eEF1G expression. Since this defect in eEF1G expression may affect its localization, we determined the intracellular localization of eEF1G in clones 1–24 and 3–9. eEF1G was similarly distributed in the cytoplasm of wild-type A549 cells and clone 3–9, whereas eEF1G was weakly detected in the cytoplasm of clone 1–24 (**Figure 1E**). We then examined whether the defect in eEF1G expression affected the other subunits of the eEF1B complex in these clones. Western blotting showed that expression of eEF1B2 and eEF1D was appreciably reduced in clones 1–24 and 3–9 (**Figure 1B**), suggesting that eEF1G may support the stability of eEF1B2 and eEF1D in cells. Taken together, these results suggest that the amount of functional eEF1B complex in clones 1–24 and 3–9 is reduced compared with that in wild-type cells.

### Clones 1–24 and 3–9 Show Normal Cell Proliferation and Protein Expression

**FIGURE 2:**
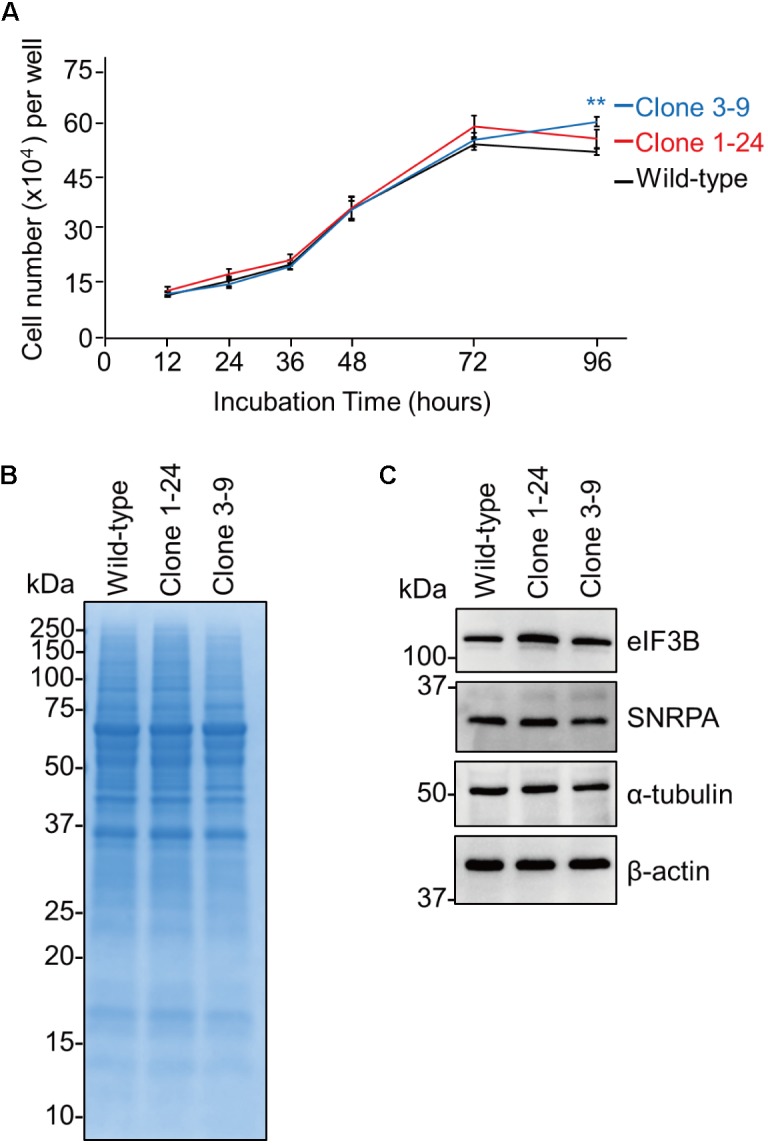
The eEF1G defect in clones 1–24 and 3–9 does not inhibit cell proliferation or endogenous protein expression. **(A)** Cell proliferation of clones 1–24 and 3–9. The data are shown as means ± SD (*n* = 3). ^∗∗^*P* < 0.01 according to a two-way ANOVA followed by Dunnett’s test. **(B)** Proteins expressed in clones 1–24 and 3–9. Total cell lysates were subjected to SDS–PAGE and visualized with CBB staining. **(C)** Expression of endogenous proteins in clones 1–24 and 3–9. Total cell lysates were analyzed by western blotting using antibodies against eIF3B, SNRPA, α-tubulin, and β-actin.

Since eEF1G is a subunit of the eukaryotic elongation factor-1 (eEF1) complex, which is responsible for the enzymatic delivery of aminoacyl-tRNAs to the ribosome (Carvalho et al., 1984) and seems to be important for cell survival (Riis et al., 1990), we evaluated the proliferation and endogenous protein expression of clones 1–24 and 3–9. To compare cell proliferation between wild-type A549 cells and the two clones, we seeded cells at a low density and counted the cell number at 12, 24, 36, 48, 72, and 96 h after seeding. We found that the number of wild-type A549 cells and the number of cells of the two clones increased at similar rates (**Figure 2A**). We next examined overall protein expression, by using CBB staining, as well as the expression of some endogenous proteins, such as eIF3B, which is involved in protein translation, SNRPA which is involved in mRNA splicing, and cytoskeletal proteins (i.e., α-tubulin and β-actin) by western blotting. We found little difference in overall protein expression or the expression of eIF3B, SNRPA, α-tubulin, and β-actin between the wild-type A549 cells and the two clones (**Figures 2B,C**). These results show that clones 1–24 and 3–9 have similar properties in terms of cell proliferation and protein expression to those of wild-type A549 cells.

### eEF1G Is Important for the Replication of WSN Virus

**FIGURE 3:**
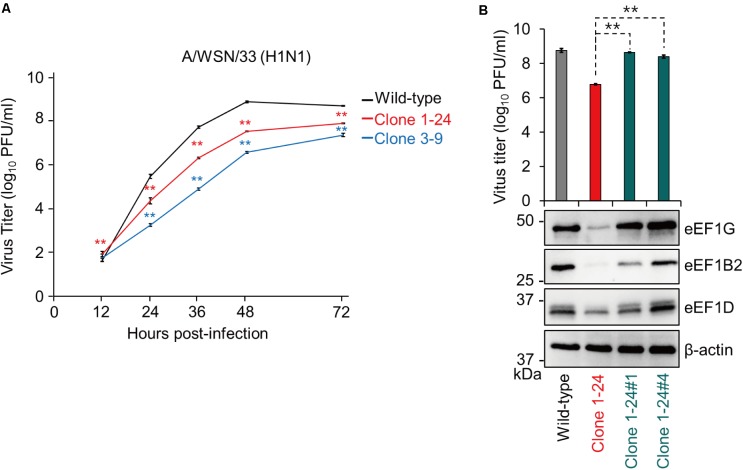
Growth of WSN virus in clones 1–24 and 3–9. Wild-type A549 cells, clone 1–24, and clone 3–9 **(A)** or wild-type A549 cells, clone 1–24, clone 1-24#1, and clone 1-24#4 **(B)** were infected with WSN virus at an MOI of 0.001. Virus titers were determined by use of plaque assays in MDCK cells. The data are shown as means ± SD (*n* = 3). ^∗∗^*P* < 0.01 according to a two-way **(A)** or one-way **(B)** ANOVA followed by Dunnett’s test. Expression of eEF1G, eEF1B2, and eEF1D was evaluated by western blotting; β-actin served as a loading control **(B)**.

We evaluated the virological significance of eEF1G during virus replication. Wild-type A549 cells, clone 1–24, and clone 3–9 were infected with WSN virus at a MOI of 0.001, and virus titers were assessed at 12, 24, 36, 48, and 72 hpi. The virus titers in the supernatant of clones 1–24 and 3–9 were significantly reduced after 24 hpi compared with those in the supernatant of wild-type A549 cells (**Figure 3A**). To validate the reduction in virus growth caused by defective eEF1G, we obtained two clones (clones 1-24#1 and 1-24#4) that stably expressed eEF1G exogenously by transfecting them with a plasmid encoding eEF1G that possessed seven synonymous mutations at the target site of gRNA1. In clones 1-24#1 and 1-24#4, expression of eEF1G, eEF1B2, and eEF1D and virus titers at 48 hpi increased compared with those in clone 1–24 but expression of eEF1B2 and eEF1D in clone 1-24#1 was lower than that in wild-type cells (**Figure 3B**). These data demonstrate that eEF1G plays an important role in the propagation of WSN virus.

### eEF1G Is Required for Viral Protein Expression

**FIGURE 4:**
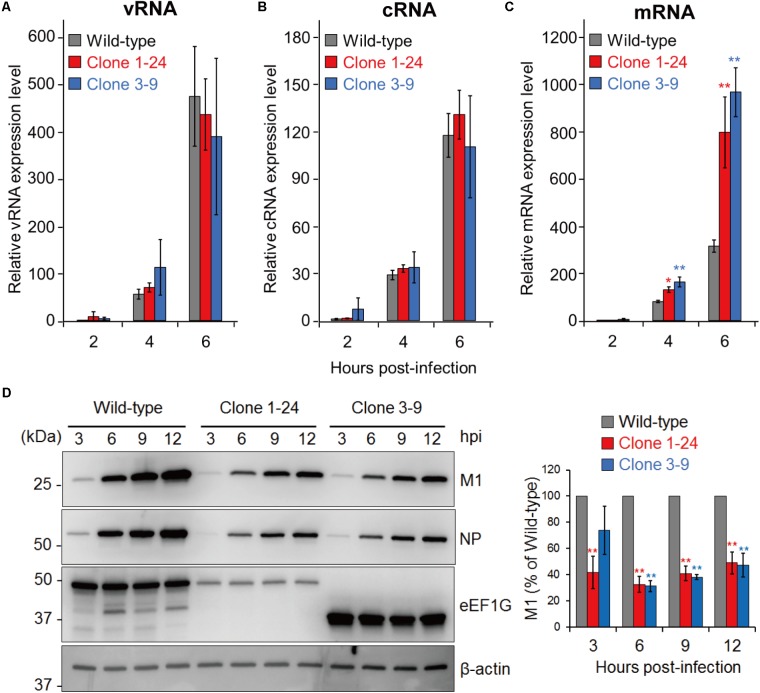
Role of eEF1G during the replication cycle of WSN virus. **(A–C)** Relative vRNA, cRNA, and mRNA expression in wild-type A549 cells, clone 1–24, and clone 3–9. The indicated cells were infected with WSN virus at an MOI of 10. Relative vRNA, cRNA, and mRNA expression was measured by strand-specific real-time RT-qPCR. The expression of vRNA, cRNA, and mRNA in wild-type A549 cells at 2 hpi was set to 1. The data are shown as means ± SD (*n* = 3). ^∗^*P* < 0.05, ^∗∗^*P* < 0.01 according to a one-way ANOVA with Bonferroni correction. **(D)** Expression of the viral proteins M1 and NP in infected clones 1–24 and 3–9. The indicated cells were infected with WSN at an MOI of 10. At 3, 6, 9, and 12 hpi, total cell lysates were analyzed by western blotting with anti-M1 and anti-NP antibodies; β-actin served as a loading control (left panel). The intensity of the M1 signals was measured and that in the wild-type A549 cells at each timepoint was set to 100% (right panel). The quantified data are shown as means ± SD (*n* = 3). ^∗∗^*P* < 0.01 according to a one-way ANOVA with Bonferroni correction.

The physiological function of eEF1G in the eEF1 complex suggests that eEF1G plays a role in the translation of viral proteins. Therefore, we examined the expression of three types of viral RNAs (vRNA, cRNA, and mRNA) and a representative viral protein (M1, which is a major component of the virion) in virus-infected cells. To compare the viral RNAs, we infected wild-type A549 cells, clone 1–24, and clone 3–9 with WSN virus at an MOI of 10, and then performed strand-specific RT-qPCR at 2, 4, and 6 hpi. In clone 1–24 and clone 3–9, expression of vRNA (**Figure 4A**), cRNA (**Figure 4B**), or mRNA (**Figure 4C**) at each timepoint was not reduced compared with that in wild-type A549 cells. To evaluate the expression of the virus protein M1, wild-type A549 cells, clone 1–24, and clone 3–9 were infected with WSN virus at an MOI of 10, and total cell lysate were prepared at 3, 6, 9, and 12 hpi. After 6 hpi, expression of M1 and NP in both infected clones was lower than that in wild-type A549 cells (**Figure 4D**). Taken together, these data indicate that eEF1G plays an important role in the translation of viral proteins.

### CA04 Virus Replicates in Clones 1–24 and 3–9 as Efficient as in Wild-Type A549 Cells

**FIGURE 5:**
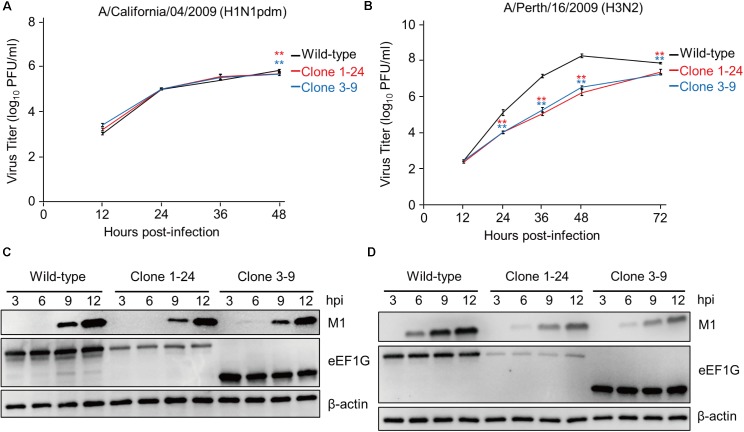
eEF1G is important for the propagation of Perth16 virus but not for CA04 virus. **(A)** Growth of CA04 virus in clones 1–24 and 3–9. The indicated cells were infected with CA04 virus at an MOI of 0.01. **(B)** Growth of Perth16 virus in clones 1–24 and 3–9. The indicated cells were infected with A/Perth/16/2009 (H3N2) virus at an MOI of 0.001. **(A,B)** Virus titers were determined by use of plaque assays in MDCK cells. The data are shown as means ± SD (*n* = 3). ^∗∗^*P* < 0.01 according to a two-way ANOVA followed by Dunnett’s test. **(C,D)** Expression of the viral protein M1 in CA04- or Perth16-infected cells. The indicated cells were infected with CA04 **(C)** or Perth16 **(D)** virus at an MOI of 10. At 3, 6, 9, and 12 hpi, total cell lysates were analyzed by western blotting with an anti-M1 antibody; β-actin served as a loading control.

To determine whether eEF1G is also important for the replication of other influenza viruses, we examined the replication of CA04 and Perth16 viruses in wild-type A549 cells, clone 1–24, and clone 3–9. These cells were infected with CA04 virus at an MOI of 0.01 or Perth16 virus at an MOI of 0.001 and virus titers were assessed at 12, 24, 36, and 48 hpi. CA04 virus replicated in clones 1–24 and 3–9 with similar efficiency to that in wild-type cells (**Figure 5A**), whereas replication of Perth16 virus in clones 1–24 and 3–9 was suppressed (**Figure 5B**). Consistent with this virus growth pattern, the expression of M1 in CA04-infected clones 1–24 and 3–9 was comparable to that in wild-type cells at 9 and 12 hpi (**Figure 5C**), whereas that in Perth16-infected clones was decreased compared with that in wild-type A549 cells (**Figure 5D**). These results suggest that the importance of eEF1G during influenza virus replication is strain specific.

### PB2 and PA Are Responsible for eEF1G Dependency

**FIGURE 6:**
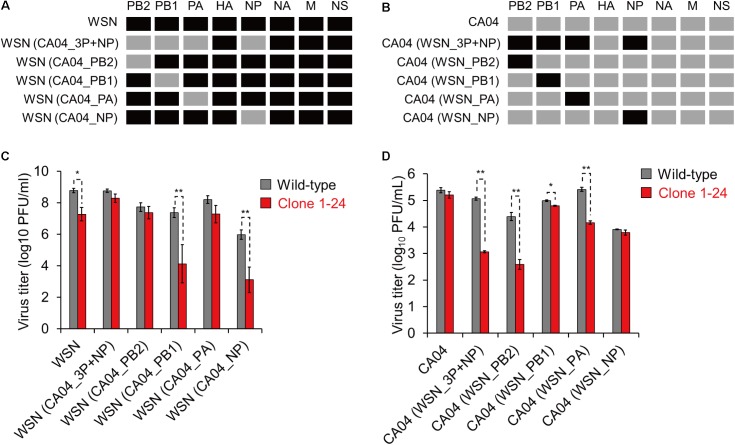
Growth of reassortant viruses in clone 1–24. **(A,B)** Schematic diagram of reassortant viruses. Black and gray indicate a viral segment derived from WSN and CA04 virus, respectively. **(C,D)** Growth of reassortant viruses in clone 1–24. Wild-type A549 cells and clone 1–24 were infected with the indicated viruses at an MOI of 0.001 for WSN and its reassortant viruses or an MOI of 0.01 for CA04 and its reassortant viruses. Virus titers at 48 hpi were determined by using plaque assays in MDCK cells. The data are shown as means ± SD (*n* = 3). ^∗^*P* < 0.05, ^∗∗^*P* < 0.01 according to a Student’s *t*-test with Bonferroni correction.

To determine which viral segment is responsible for the difference in eEF1G-dependence among virus strains, we prepared two reassortant viruses: WSN (CA04_3P+NP), which contained PB2, PB1, PA, and NP from CA04 and the rest of its gene segments from WSN; and CA04 (WSN_3P+NP), which contained PB2, PB1, PA, and NP from WSN and the rest of its gene segments from CA04. Since eEF1G was involved in viral protein translation and was previously identified as an interacting partner of PB2, PB1, PA, and NP derived from WSN virus (Watanabe et al., 2014), the reassortant viruses were prepared by exchanging the PB2, PB1, PA, and NP segments between the WSN and CA04 viruses (**Figures 6A,B**). Then, these two reassortant viruses together with their parental WSN and CA04 viruses were inoculated into wild-type A549 cells and clone 1–24 and virus titers were assessed at 48 hpi. Although the virus titers of the tested viruses varied in the wild-type A549 cells, the titers of the WSN and CA04 (WSN_3P+NP) viruses in clone 1–24 were significantly reduced, whereas those of the CA04 and WSN (CA04_3P+NP) viruses in clone 1–24 were not (**Figures 6C,D**). These results suggest that the PB2, PB1, PA, and NP segments of WSN virus are primarily responsible for its eEF1G-dependent replication.

Next, we generated four single-segment reassortant viruses in the background of the WSN (**Figure 6A**) or CA04 segments (**Figure 6B**), and compared their growth properties in clone 1–24. The WSN (CA04_3P+NP), WSN (CA04_PB2), and WSN (CA04_PA) viruses replicated in clone 1–24 with similar efficiency as that in wild-type A549 cells (**Figure 6C**), whereas the growth of the WSN, WSN (CA04_PB1), and WSN (CA04_NP) viruses in clone 1–24 was decreased compared with that in wild-type cells (**Figure 6C**). The opposite results were obtained when the experiments were done in the background of the CA04 segments, except that the replication of CA04 (WSN_PB1) virus in clone 1–24 was slightly, but significantly, reduced (**Figure 6D**). These results suggest that PB2 and PA derived from WSN play major roles in eEF1G-dependent virus replication.

## Discussion

Our group previously found that eEF1G is one of the host proteins involved in the replication cycle of influenza A virus by analyzing the interactome between WSN virus and the host proteins in human embryonic kidney 293 cells (Watanabe et al., 2014). Here, we showed that eEF1G also played an important role in the replication of WSN virus in human alveolar adenocarcinoma epithelial A549 cells. Since eEF1G is a subunit of the eukaryotic elongation factor-1 (eEF1) complex, which is responsible for the enzymatic delivery of aminoacyl-tRNAs to the ribosome (Carvalho et al., 1984; Riis et al., 1990), we speculated that eEF1G may be important for the replication of all types of influenza A virus. However, eEF1G deficiency did not affect the replication of CA04 virus, implying that influenza viruses differ in their use of host proteins during replication. Since seven of the eight genome-wide screens that have been conducted to date used similar viruses (WSN or PR8 virus) (Brass et al., 2009; Shapira et al., 2009; Karlas et al., 2010; König et al., 2010; Ward et al., 2012; Su et al., 2013; Tran et al., 2013; Watanabe et al., 2014), screenings using other viruses, such as CA04 virus, may identify additional host proteins and reveal novel insights into influenza virus replication.

WSN virus reduced virus protein expression in both clone 1–24 and clone 3–9. Clone 1–24 showed decreased eEF1G expression due to one amino acid substitution and a 4-amino acid deletion, whereas clone 3–9 expressed smaller eEF1G than authentic eEF1G in wild-type A549 cells because of a frameshift. In clone 1–24, the decreased eEF1G expression reduced the amount of eEF1B complex, which is a heterotrimer of eEF1B2, eEF1D, and eEF1G and possesses guanine nucleotide exchange activity to activate eEF1A (Janssen and Möller, 1988). Downregulation of the eEF1B complex might cause a reduction in active eEF1A, which delivers aminoacyl-tRNAs to the ribosome, resulting in lower viral protein expression. In clone 3–9, the shorter form of eEF1G, which lacks the C-terminal region of authentic eEF1G, was expressed at a comparable level to that of authentic eEF1G in wild-type A549 cells and expression of eEF1B2 and eEF1D was decreased. This finding suggests that the C-terminus region of eEF1G is functionally important for virus protein expression via maintenance of eEF1B2 and eEF1D expression. eEF1G has two domains: the N-terminal glutathione-*S*-transferase (GST) domain (Koonin et al., 1994) and the C-terminal highly conserved eEF1G domain (Gillen et al., 2008; Fan et al., 2010). The N-terminal GST domain is responsible for the interaction with both eEF1B2 and eEF1D (Mansilla et al., 2002), whereas the C-terminal eEF1G domain possesses a putative conserved phosphorylation site, which is essential for the activity of the eEF1B complex in *Drosophila* (Fan et al., 2010). Deletion of the C-terminal eEF1G domain thus seems to reduce the amount of active eEF1B complex, resulting in a decrease in active eEF1A. Since eEF1A is also involved in WSN virus replication in A549 cells (Karlas et al., 2010), we suggest that eEF1G contributes to the translation of virus proteins as a member of the eEF1 complex.

In our study, eEF1G-dependent replication was attributed to the PB2 and PA of WSN virus. Subunits of the eEF1 complex have been reported to support the replication of various viruses via interactions with viral proteins or viral genomic RNA (Li et al., 2013). For example, eEF1A, eEF1D, and eEF1G have been shown to be required for the polymerase activity of vesicular stomatitis virus (VSV), which possesses a negative single-stranded genomic RNA, via an interaction with RNA-dependent RNA polymerase L (Das et al., 1998). Similarly, the interaction of eEF1A and eEF1G with the reverse transcriptase of HIV-1 stabilizes the reverse transcription complex of HIV (Warren et al., 2012). Taken together, these previous reports and our current results suggest the possibility that eEF1G and the other subunits of the eEF1 complex may also have a non-canonical role, such as serving as a scaffold for the functions of the virus polymerase via specific interactions with PB2 and/or PA in influenza virus-infected cells. The interaction of eEF1G with the virus polymerase proteins of WSN and Perth16 viruses may differ from that with CA04 virus. Therefore, further studies focused on mechanistic aspects are required and would help us to understand the direct roles of eEF1G in virus propagation.

In summary, here we found that eEF1G is involved in the translation of viral proteins in WSN- and Perth16-infected cells but not in CA04-infected cells. Further studies are needed to understand the roles of eEF1G during viral protein synthesis.

## Author Contributions

SS and SY designed the study. SS performed the experiments. SS, SY, and YK analyzed the data and wrote the manuscript. All authors reviewed and approved the manuscript.

## Conflict of Interest Statement

YK has received speaker’s honoraria from Toyama Chemical and Astellas, Inc., and has received grant support from Chugai Pharmaceuticals, Daiichi Sankyo Pharmaceutical, Toyama Chemical, Tauns Laboratories, Inc., Otsuka Pharmaceutical, Co., Ltd., and Denka Seiken, Co., Ltd., and is a co-founder of FluGen. The remaining authors declare that the research was conducted in the absence of any commercial or financial relationships that could be construed as a potential conflict of interest.

## Footnotes

1 https://portals.broadinstitute.org/gpp/public/
